# A Rare Case of Pleural Epithelioid Mesothelioma With a Prominent Myxoid Stroma Reported With Morphology, Fluorescent In Situ Hybridization, and Ultrastructural Findings

**DOI:** 10.7759/cureus.62212

**Published:** 2024-06-12

**Authors:** Hiroshi Sonobe, Rika Omote, Toshiyuki Habara, Kazuhiro Washio, Nobuyoshi Yamazoe, Shinji Matsumoto, Kazuki Nabeshima, Hiroko Toda

**Affiliations:** 1 Department of Diagnostic Pathology, National Hospital Organization Fukuyama Medical Center, Fukuyama, JPN; 2 Department of Clinical Laboratory, Chugoku Central Hospital of the Mutual Aid Associations of Public School Teachers, Fukuyama, JPN; 3 Department of Surgery, Chugoku Central Hospital of the Mutual Aid Associations of Public School Teachers, Fukuyama, JPN; 4 Department of Internal Medicine, Mitama Clinic, Fuchu, JPN; 5 Department of Pathology, Fukuoka University Hospital, Fukuoka, JPN; 6 Department of Diagnostic Pathology, Pathological Diagnosis Center, Fukuoka Tokushukai Hospital, Fukuoka, JPN; 7 Department of Diagnostic Pathology, Chugoku Central Hospital of the Mutual Aid Associations of Public School Teachers, Fukuyama, JPN

**Keywords:** ultrastructure, fluorescence in situ hybridization, immunohistochemistry, myxoid stroma, epithelioid mesothelioma

## Abstract

Herein, we report a rare case of pleural epithelioid malignant mesothelioma with a prominent myxoid stroma. To date, detailed morphological or molecular pathological findings have not been reported for this type of tumor. Hence, we aimed to describe the cytological, histological, immuno-cytohistological, electron-microscopic, and molecular pathological findings using fluorescence in situ hybridization (FISH) in such a case. The patient was a male in his mid-sixties with a history of asbestos exposure and had originally visited the hospital with a persistent cough and fever. Chest radiography revealed left pleural effusion, and laboratory examination revealed a high titer for hyaluronic acid in the effusion. Additionally, computed tomography revealed diffuse multinodular or cystic lesions in the left parietal pleura, and pleural effusion cytology revealed large epithelioid cells with mild nuclear atypia, which were considered reactive mesothelial cells. Cytologically, Giemsa staining revealed that these cells harbored variously sized intracytoplasmic vacuoles that were Alcian-blue-positive, suggesting hyaluronan production. Biopsy revealed large epithelioid cells that loosely proliferated against a prominent myxoid background. These cells were immuno-positive for calretinin, Wilms’ tumor 1, D2-40, vimentin, and cytokeratin AE1/AE3 but not for carcinoembryonic antigen, Ber-EP4, or desmin. BRCA 1 associated protein 1 immunostaining showed nuclear loss, and FISH showed homozygous deletion of cyclin-dependent kinase inhibitor 2A (p16) on chromosome 9p21. Based on these findings, the lesion was diagnosed as an epithelioid mesothelioma with a prominent myxoid stroma. Electron-microscopy demonstrated a dense microvillus pattern on the surface of the tumor cells, indicating a mesothelial cell origin, and variously sized vacuoles in the cytoplasm, confirming the presence of intracytoplasmic vacuoles demonstrated on cytology. The tumor tissues obtained during surgery harbored prominent myxoid stroma, which proved that the present tumor was consistent with this type of mesothelioma. After informed consent was obtained, the patient and family wished for total resection of the tumor and postoperative chemotherapy, and the patient eventually died eight months after surgery.

## Introduction

As relatively rare tumors, mesotheliomas are related to a patient’s history of asbestos exposure in more than 80% of tumors [1]. In such cases, asbestos fibers induce abnormal changes of cyclin-dependent kinase inhibitor 2A (CDKNA2A) [2]. However, mesotheliomas are also found in patients with an uncertain history of asbestos exposure [3]. Mesotheliomas arise from mesothelial cells in the thoracic, abdominal, and pericardial cavities, as well as the tunica vaginalis testis. More than 80% of mesotheliomas develop in the thoracic cavity, with approximately 10% to 15% found in the peritoneal cavity, and less than 1% in the pericardial cavity or tunica vaginalis testis [4].

According to the World Health Organization (WHO) classification (2015, 4th edition) [4], diffuse pleural mesotheliomas can be classified histologically into three subtypes: epithelioid, sarcomatoid, and biphasic. Many variants and patterns, such as solid, tubulo-papillary, trabecular, micropapillary, adenomatoid, clear cell, transitional, deciduoid, myxoid, pleomorphic, signet ring cell, lympho-histiocytoid, and small cell, exist within the epithelioid subtype, reflecting its histological diversity. Nuclear grading of tumors has also been established. Most variants and patterns have an unfavorable prognosis, resulting in a median survival of 8 to 12 months if untreated and a five-year survival rate of 5% [5]. However, the myxoid variant is an exception with a favorable prognosis [6,7]. In the new WHO classification (2021, 5th edition) [8], which was published after summarizing the contents of the previous classification (2015, 4th edition) and subsequent significant information, two categories are identified: one is pre-invasive mesothelial tumors, including well-differentiated papillary mesothelial tumors, mesothelioma in situ, and adenomatoid tumors, and another is diffuse mesothelioma. In the new classification, the evaluation of prognoses, favorable or unfavorable, focuses on structural patterns, cytologic findings, and stromal features.

To date, few reports have described the morphological and molecular findings of the myxoid variant or mesothelioma with a prominent myxoid stroma. More than 10 years prior to this report, we encountered a rare case of pleural epithelioid mesothelioma with a prominent myxoid stroma. We believe that it is worthwhile to describe the cytological, histopathological, molecular or genetic, and ultrastructural characteristics of this tumor type. Thus, the purpose of the report is to describe the detailed characteristics of the tumor in such a case, along with associated clinical findings.

## Case presentation

**Figure 1:**
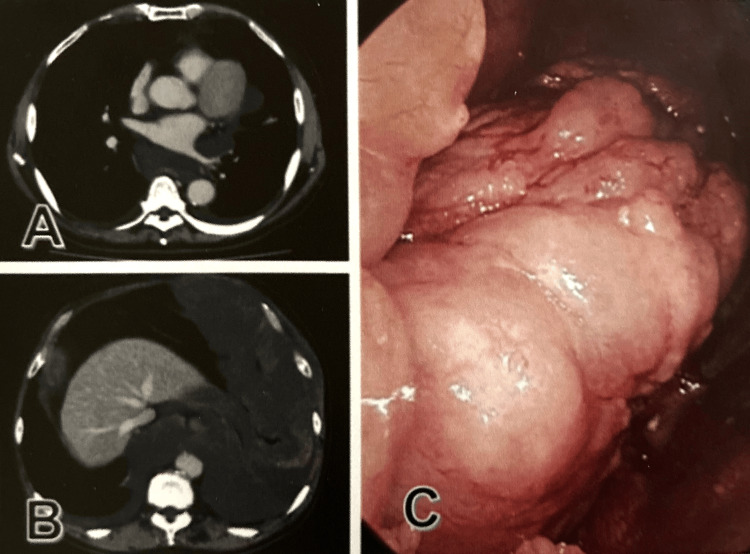
CT and endoscopic findings A-C: A multinodular cystic lesion widely developing on the pleura from the mediastinum to the diaphragm (A, B: CT; C: endoscopy). CT: computed tomography

The patient in this case was a male construction worker in his mid-sixties with a history of asbestos exposure. Because of a lingering cold, he visited a family doctor with a persistent cough and fever, and chest radiography revealed left pleural effusion. One month later, the patient was referred to Chugoku Central Hospital. On laboratory examination, the serum tumor markers including carcinoembryonic antigen (CEA), cytokeratin 19 fragment, and α-fetoprotein were within normal limits. Hyaluronic acid was abnormally elevated (>200,000 ng/ml) in the pleural effusion. Computed tomography and endoscopy revealed multinodular or cystic lesions in the left parietal pleura of the mediastinum and diaphragm (Figure 1).

Subsequently, pleural effusion cytology and tumor biopsy were performed. Based on the results, the patient was diagnosed as having epithelioid mesothelioma with a prominent myxoid stroma. A scrupulous explanation of this disease was provided to the patient and his family. According to their wishes, the lesion was totally excised. Thereafter, the first course of chemotherapy comprising carboplatin and pemetrexed sodium hemipentahydrate was initiated, and the second one was performed three weeks later (Table 1).

**Table 1:** Composition of chemotherapy in a course AUC: area under the curve

Anti-cancer agent	Course
carboplatin	4.5 (AUC）
pemetrexed sodium hemipentahydrate	500 (mg/m^2^）

**Figure 2:**
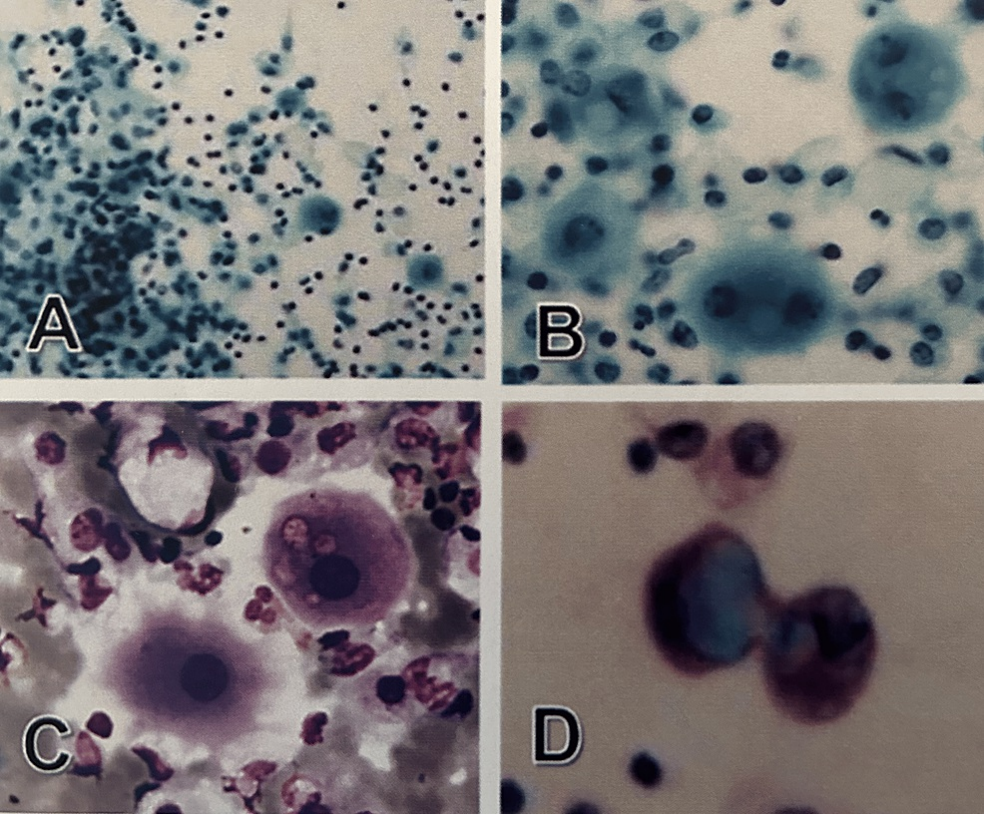
Pleural effusion cytology A-D: Large epithelioid cells with mild nuclear atypia appear together with various inflammatory cells (A, B: Papanicolaou). The large epithelioid cells have intracytoplasmic vesicles to vacuoles (B: Papanicolaou; C: Giemsa) and are positive for Alcian-blue (D). A: Original magnification x40; B-D: Original magnification x400.

However, after no effect on progressive disease was observed, chemotherapy was discontinued, and the patient died eight months after surgery. Cytological examination of the pleural effusion revealed large, round epithelioid cells scattered as individuals or small clusters against the background of inflammatory findings, including lymphocytes and macrophages. On Papanicolaou staining, these large epithelioid cells uniformly stained pale green and contained cytoplasm with variously sized vacuoles positive for Alcian-blue stain. Mildly atypical, rounded nuclei were observed in the central part of the cytoplasm (Figure 2). Based on these findings, we considered that these cells were reactive mesothelial cells rather than neoplastic cells.

**Figure 3:**
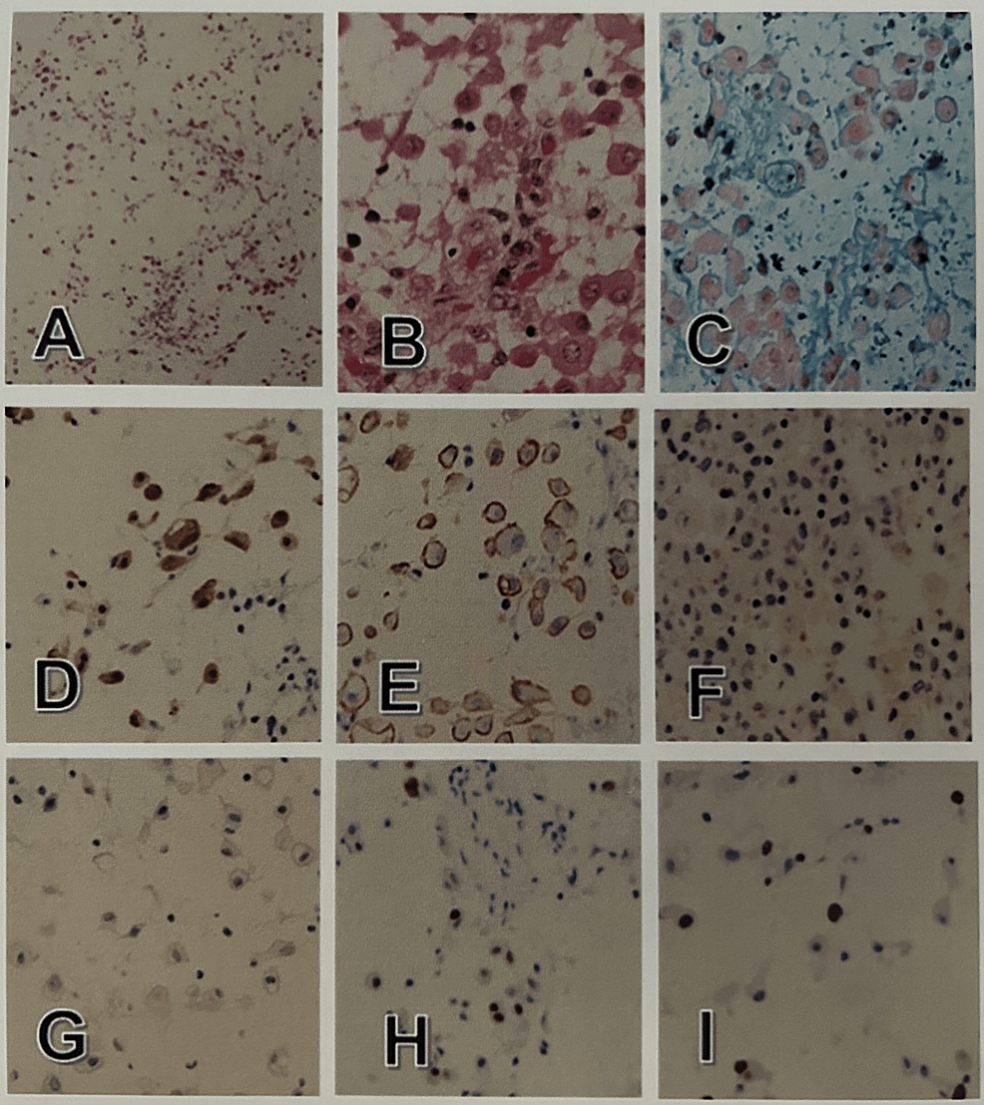
Histological and immuno-histochemical features A-C: Mildly atypical large epithelioid cells loosely proliferate with prominent myxoid background (A, B: hematoxylin-eosin; C: Alcian-blue). (A) Original magnification x40. (B, C) Original magnification x200. These cells are immuno-positive for calretinin (D) and D2-40 (E) but not for Ber-EP4 (F) or CEA (G), with a Ki-67 index of 10 (H) and p53 positivity of 20% (I). D-I: Original magnification x100. CEA: carcinoembryonic antigen

Biopsy revealed large, round epithelioid cells with abundant eosinophilic cytoplasm and round nuclei with mild atypia, and these cells scattered with low cell density with prominent myxoid stroma found throughout the tissue. No necrotic, hemorrhagic, or obvious inflammatory foci were observed, and nuclear mitotic figures were barely seen. Immunohistochemically, the large epithelioid tumor cells were positive for calretinin, D2-40, WT1, EMA, CD146, cytokeratin AE1/AE3 (AE1/AE3), and vimentin but not for CEA, Ber-EP4, or desmin. These cells exhibited positive reactions for p53 (10%) and Ki67 (index 20) (Figure 3, Table 2) and nuclear negativity for BRCA1-associated protein 1 gene (BAP-1). Based on these findings, the histological diagnosis was malignant mesothelioma of the myxoid variant according to the WHO classification system (2015, 4th edition).

**Table 2:** Tumor cell immunostaining results WT-1: Wilms tumor 1; EMA: epithelial membrane antigen; AE1/AE: cytokeratin AE1/AE3; CEA: carcinoembryonic antigen; BAP1: BRCA1 associated protein-1

Antibody	Tumor tissue	Antibody	Tumor tissue
Calretinin	Positive	CEA	Negative
WT1	Positive	BER-EP4	Negative
D2-40	Positive	Desmin	Negative
EMA membranous	Positive	CD68	Negative
CD146	Positive	BAP1 nuclear loss	Yes
Vimentin	Positive	Ki-67	Index: 10
AE1/AE3	Positive	p53	Positivity: 20%

**Figure 4:**
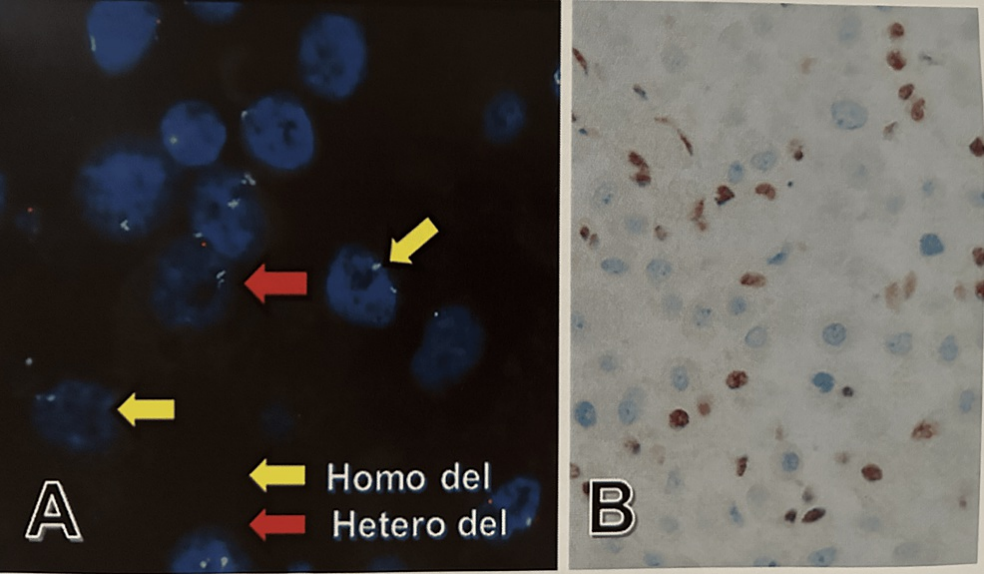
CDKN2A (p16)-FISH and BAP1 immunohistochemistry A: p16-FISH reveals homozygous deletion (yellow arrow) and heterogenous deletion (red arrow) in tumor cells. B: Tumor cells show nuclear loss of BAP1, and non-tumor cells reveal nuclear positivity for BAP1; Original magnification x200. CDK2A: cyclin-dependent kinase inhibitor 2A; FISH: fluorescence in situ hybridization; BAP1: BRCA1-associated protein-1

Fluorescence in situ hybridization (FISH) performed using Agilent probe of cyclin-dependent kinase inhibitor 2A (CDKN2A) (p16) on chromosome 9p21 revealed 25.4% (15/59) of homozygous deletion and 49.2% (29/59) of heterozygous deletions, and BAP1-immunohistochemistry revealed nuclear negativity in tumor cells (Table 3, Figure 4).

**Table 3:** CDKN2A (p16)-fluorescence in situ hybridization results FISH: fluorescence in situ hybridization; CDKN2A: cyclin-dependent kinase inhibitor 2A

CDK2A(p16)-FISH	Agilent probe
Homogenous deletion	25.4% (15/59)
Heterogenous deletion	49.2% (29/59)
Normal pattern	25.4% (15/59)

**Figure 5:**
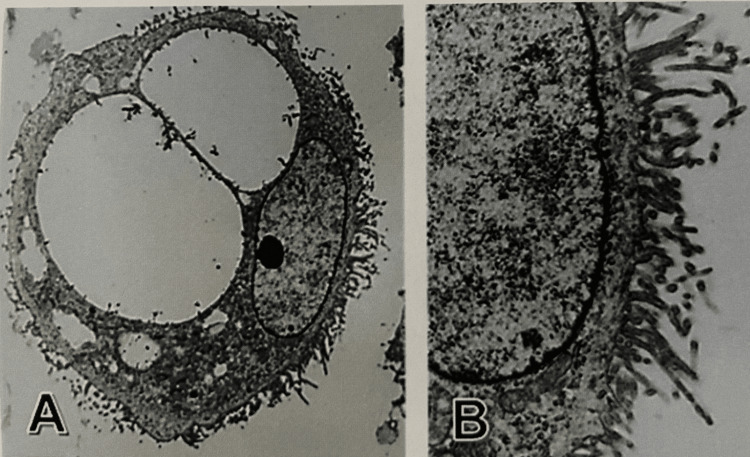
Ultrastructural features A: The tumor cell possesses increased euchromatin and distinct nucleoli with intracytoplasmic small vesicles and large vacuoles. B: The cell surface reveals a characteristic microvillous pattern. (A) Original magnification x3,000; (B) Original magnification x9,000.

Ultra-microscopically, the tumor cells exhibited ovoid nuclei with increased euchromatin, large nucleoli, and vesicles of various sizes in the cytoplasm. Additionally, the cell surface was highly microvillus (Figure 5). The above-mentioned FISH and electron-microscopic findings supported the view that the tumor was a mesothelioma. Histological examination of several sections of the surgical material revealed similar to those observed on biopsy.

## Discussion

The most recent WHO classification guidelines for diffuse pleural epithelioid mesothelioma (2021, 5th edition) [8] focused on structural patterns, cytologic findings, stromal features, and prognosis. This is because uniform epithelioid tumor cells without noticeable atypia typically exhibit a tubulo-papillary pattern in the pleura in the early phase. Thereafter, as the tumor cells proliferate, invade, and develop further, the structural patterns and cytological characteristics usually change, as the cell diversifies. Therefore, histologically, the grade (low to high) as well as the various structural patterns and their percentages are important for tumor prognoses. Tubulo-papillary, trabecular, and adenomatoid of structural patterns, myxoid stroma in > 50% of tumors and lympho-histiocytoid tumors are associated with a favorable prognosis. Conversely, solid or micropapillary patterns in >50% of tumors suggest an unfavorable prognosis. Nuclear atypia is graded as 1 for mild, 2 for moderate, and 3 for high; mitotic counts are categorized as low (1 point at 1 mitosis/2 mm^2^ or less), intermediate (2 points at 2-4 mitosis/2 mm^2^), and high (3 points at 5 mitosis/2 mm^2^ or more). A higher nuclear degree in tumors leads to a poorer prognosis. The presence of rhabdoid cells, pleomorphic cells, and high nuclear grade in tumors leads to a poor prognosis. The overall tumor score is determined based on the nuclear grade and necrosis combined with the presence or absence; thus, the scores are defined as low for nuclear grades I and II without necrosis and high for nuclear grade III and nuclear grade II with necrosis. Additionally, BAP1, enhancer of zeste 2 Polycomb progress complex 2 subunit, and S-methyl-5’-thioadenosine phosphorylase (MTAP) loss detected by immunohistochemistry, or CDKN2A (p16) homozygous deletion by FISH is used for differentiation between benign mesothelial proliferation and mesothelioma. The biphasic subtype is classified based on a minimum of more than 10% of either epithelioid or sarcomatoid components; this is the same criterion as that in the previous WHO classification (2015, 4th edition) in cases of resection specimens, but this 10% rule is not applicable to biopsy or cytological specimens [9-11].

Distinguishing whether a certain pleural change corresponds to epithelioid mesothelioma; reactive mesothelial proliferation; fibrous pleuritis; various carcinomas, including lung cancer and cancer metastases; and mesenchymal tumors can be difficult. In such cases, molecular and genetic studies are useful for diagnosis [11-13]. Genetic alterations in mesothelioma commonly include BAP1, CDKN2A, and moesin-ezrin-radixin-like tumor suppressors, which play important roles in mesothelioma development [14]. BAP1 mutations are the most common and occur as early events, with BAP1 deficiency resulting in somatic biallelic mutations that lead to tumorigenesis, as BAP1 regulates chromatin-related processes, such as gene expression, deoxyribonucleic acid (DNA) replication, and DNA repair [15]. Immunohistochemical loss of BAP1 expression is less sensitive (42%-65%) but 100% specific. Therefore, it is extremely useful in differentiating between mesothelioma and reactive mesothelial proliferation [16]. CDKN2A abnormalities are confirmed by FISH, and loss of MTAP expression, by immunohistochemistry. The sensitivity for MTAP loss is modest (42%-48%), but the specificity is 100%. Therefore, CDKNA 9p21 evaluated by FISH and MTAP evaluated by immunohistochemistry are also useful for the diagnosis of malignant mesothelioma [17,18]. The reliability of diagnosis can be increased using both the BAP1 and MTAP staining methods [19].

As the pleural tumor in the present case exhibited immunohistochemical findings supporting a diagnosis of mesothelioma and as all tumor tissue obtained from the biopsy and surgery consisted of abundant myxoid stroma, the patient was diagnosed with epithelioid mesothelioma with a prominent myxoid stroma. Applying the criteria for prognostic factors described in the WHO classification (2021, 5th edition) [8], this tumor was evaluated as follows: nuclear grade I, low mitotic figure, no necrosis, and low overall tumor score. In addition, it was evaluated to have a favorable prognosis because of the abundant myxoid stroma. However, after informed consent was obtained, the patient and family wished for total resection of the tumor and chemotherapy, and the patient eventually died eight months after the surgery. On the other hand, we were able to find an autopsy report on this type of tumor in the literature [20]. An elderly woman with no history of asbestos exposure was diagnosed with pleural epithelioid mesothelioma with a prominent myxoid stroma on biopsy. The patient chose not to undergo aggressive surgery or chemotherapy after informed consent was obtained. Thereafter, she survived for more than five years after diagnosis. An autopsy revealed that the tumor occupied the entire pleural space on the affected side and highly compressed the lung; however, no pulmonary invasion or distant metastasis was present. The histology of the tumor was quite identical to that at biopsy, with no higher-grade components, such as rhabdoid cells, pleomorphic cells, or high-grade nuclear atypia. This case provides a valuable illustration of the natural progression of this type of tumor. To date, the above-mentioned molecular examinations on the epithelioid mesothelioma with a prominent stroma have scarcely been reported. In the present case, cytology revealed that the mesothelioma cells should be considered reactive owing to unremarkable nuclear atypia. However, BAP1 immunostaining demonstrated nuclear loss and p16-FISH demonstrated homogenous deletion in tumor cells, confirming a diagnosis of mesothelioma.

## Conclusions

The tumor in this case was a rare pleural diffuse epithelioid mesothelioma with a prominent myxoid stroma. To date, there have been few reports, including molecular and genetical studies, on this type of tumor. In the present case, cytology revealed that the mesothelioma cells should be considered reactive owing to unremarkable nuclear atypia. However, BAP1 immunostaining demonstrated nuclear loss and p16-FISH demonstrated homogenous deletion in tumor cells, confirming that the tumor was a mesothelioma. An accumulation of cases is necessary to elucidate the characteristics of this type of mesothelioma in detail.
